# Assessment of Duplicate Publication of Chinese-Sponsored Randomized Clinical Trials

**DOI:** 10.1001/jamanetworkopen.2020.27104

**Published:** 2020-12-03

**Authors:** Yuanxi Jia, Doudou Huang, Jiajun Wen, Riaz Qureshi, Yehua Wang, Lori Rosman, Qingkun Chen, Karen A. Robinson, Joel J. Gagnier, Stephan Ehrhardt, David D. Celentano

**Affiliations:** 1 Bloomberg School of Public Health, The Johns Hopkins University, Baltimore, Maryland; 2 Welch Medical Library, The Johns Hopkins University, Baltimore, Maryland; 3 Institute of Medical Information and Medical Library, Chinese Academy of Medical Sciences and Peking Union Medical College, Beijing, China; 4 School of Medicine, The Johns Hopkins University, Baltimore, Maryland; 5 Michigan Medicine, School of Public Health, University of Michigan, Ann Arbor

## Abstract

**Importance:**

Duplicate publications of randomized clinical trials are prevalent in the health-related literature. To date, few studies have assessed the interaction between duplicate publication and the language of the original publication.

**Objective:**

To assess the existence of duplicate publication and the extent to which duplicate publication is associated with the language of the original publication.

**Design, Setting, and Participants:**

In this retrospective cohort study, eligible randomized clinical trials were retrieved from trial registries, and bibliographic databases were searched to determine their publication status. Eligible randomized clinical trials were for drug interventions from January 1, 2008, to December 31, 2014. The search and analysis were conducted from March 1 to August 31, 2019. The trial registries were either primary registries recognized by the World Health Organization or the Drug Clinical Trial Registry Platform sponsored by the China Food and Drug Administration.

**Exposures:**

Individual randomized clinical trials with positive vs negative results.

**Main Outcomes and Measures:**

Journal articles were classified as main articles (determined by largest sample size and longest follow-up among all journal articles derived from that randomized clinical trial) and duplicates. The duplicates were classified into 4 types: (1) unreferenced subgroup analysis (article did not disclose itself as a subgroup analysis or reference its main article); (2) unreferenced republication (article did not disclose itself as a replicate of the main article or reference it); (3) unreferenced interim analysis (article did not disclose itself as an interim analysis or reference its main article); and (4) partial duplicate (article did not disclose its sharing a subset of participants with other articles or reference them).

**Results:**

Among 470 randomized clinical trials published by August 2019 as journal articles, 55 (11.7%) had 75 duplicates, of which 53 (70.7%) were cross-language duplicates. Of the 75 duplicates, 33 (44.0%) were unreferenced republications, 25 (33.3%) unreferenced subgroup analyses, 15 (20.0%) unreferenced interim analyses, and 2 (2.7%) partial duplicates. When the main article of a randomized clinical trial was published in Chinese, those with positive findings were 2.48 (95% CI, 1.08-5.71) times more likely to have subsequent duplicate publication than those with negative findings.

**Conclusions and Relevance:**

In this study, most duplicates were cross-language duplicates and the most common type was unreferenced republication of the main article. Duplicate publication bias exists when the main articles of randomized clinical trials were published in Chinese, potentially misleading readers and compromising journals and evidence synthesis.

## Key Points

**Question:**

Are publication language (English and Chinese) and trial results associated with duplicate publication of randomized clinical trials conducted in mainland China?

**Findings:**

This cohort study included 470 Chinese-sponsored randomized clinical trials identified from clinical trial registries and published between 2008 and 2019. Approximately 12% of those randomized clinical trials have at least 1 duplicate publication; most are published in a different language than the main article. When main articles are published in Chinese, those with positive findings are more likely to have subsequent duplicate publication than those with negative findings.

**Meaning:**

Results of this study suggest that publishing randomized clinical trials in different languages may impede identification of duplicates and be associated with duplicate publication bias.

## Teaser

This cohort study of Chinese-sponsored randomized clinical trials assesses incidence of duplicate publication and the extent to which duplicate publication is associated with the language of publication.

## Introduction

A duplicate publication (referred to as a duplicate) “overlaps substantially with one already published, without clear, visible reference to the previous publication.”^1^ Duplicates waste resources, breach copyright, undermine the integrity of research, and distort evidence if included in meta-analyses and systematic reviews.^2,3,4^

When disseminating substantial information, secondary publications across languages can be justified or even recommended to maximize audience and potential reach.^1^ Such secondary publications should appropriately disclose the relationship to the main publication to avoid being treated as an independent study.^1^

Duplicates are prevalent in the health-related literature.^5,6,7,8,9,10^ The challenge remains, however, for readers, journal editors, and meta-analysts to detect duplicates, especially when the authorship, design, or results of duplicates deviate from those of the main publication.^11^ Moreover, duplicate status may be concealed by language barriers that increase the challenge of detection. For example, English-speaking peer reviewers and editors may be unlikely to detect a duplicate of a non-English original publication.

To our knowledge, few studies have assessed the interaction between duplicates and the language of publication. How often cross-language duplicates occur or the role of language in the production of duplicates and subsequent biasing of evidence syntheses is unclear. We selected randomized clinical trials (RCTs) from mainland China as an example for our study for several reasons: first, the biomedical research community is witnessing a proliferation of RCTs from mainland China^12,13^; second, most Chinese literature is not indexed in English bibliographic databases and a shift toward publishing RCTs from mainland China in English has not been observed^14,15^; third, the coverage of the Chinese literature by English bibliographic databases has been gradually increasing^16,17^; and fourth, the Chinese literature and bibliographic databases have been increasingly recognized by the evidence synthesis community.^18^ The aims of this study were to estimate prevalence and detect types of duplicates among RCTs from mainland China, and to evaluate the existence of duplicate publication bias with a focus on the interaction between duplication and language of publication.

## Methods

In this cohort study, we retrieved eligible RCTs from trial registries, identified the corresponding journal articles (including duplicates) from bibliographic databases, and evaluated duplicate types and duplicate publication bias. This study followed the Strengthening the Reporting of Observational Studies in Epidemiology (STROBE) reporting guideline for cohort studies.^19^ The study was not subject to institutional review board approval because all the data were open source and no participants were involved (per US Department of Health and Human Services regulation under the 45 CFR 46 Common Rule).

### Identifying RCTs From Trial Registries and Databases

Our study sample comprised RCTs sponsored by organizations located in mainland China. The process to retrieve registry records from trial registries and the corresponding journal articles from bibliographic databases has been described previously.^20^ Briefly, we considered RCTs to be eligible if they evaluated the efficacy and/or safety of drugs and were conducted between January 1, 2008, and December 31, 2014. We considered a substance to be a drug if recognized and regulated by the US Food and Drug Administration and/or the European Medicine Agency.^21,22^ We excluded bioequivalence studies, pharmacokinetics studies, and RCTs with missing information on eligibility of participants, study period, experimental drugs, principal investigator, or sponsor.

We included only journal articles originating from eligible RCTs. The search strategy and terms updated from previous studies,^23,24,25^ list of bibliographic databases,^26^ and criteria to match journal articles with registry records are presented in the eMethods, eTable 1, eTable 2, and eTable 3 in the Supplement. An example of literature search can be found in the eAppendix in the Supplement. Two of us (Y.J. and D.H.) independently searched bibliographic databases and identified the corresponding journal articles of eligible RCTs. Discrepancies were discussed and resolved with a third author (J.W.).

### Identifying the Main Article and Duplicates

We defined the main article for an RCT as the study with the largest sample size and the longest follow-up among all journal articles derived from that RCT.^11^ If multiple articles existed with the same sample size and follow-up period, we considered whichever was submitted earliest to an academic journal as the main article. We operationally defined a duplicate as an article that had at least 1 identical outcome but did not reference its relationship to the main article.

In this study, we focused on duplicates that might distort evidence; therefore, although article elements such as background, methods, or discussion were used in the identification of duplicates, we only considered duplicate outcomes for analyses. We categorized outcomes into 5 elements: domain (eg, systolic blood pressure), specific measurement (eg, a device at sitting position), specific metric (eg, change from baseline), the method of aggregation (eg, mean/median), and time point.^27^ An outcome was considered duplicated if it had identical domain, specific measurement, and time point sets with an outcome in the main article, regardless of the specific measure or the method of aggregation. For example, we considered an outcome measuring systolic blood pressure a duplicate if it reported the mean systolic blood pressure while the main article reported the median, as long as the method of measurement and time point were consistent.

### Duplicate Types

We classified duplicates into 4 types based on the progress of recruitment and follow-up of the RCTs: (1) unreferenced subgroup analyses; (2) unreferenced republications; (3) unreferenced interim analyses; and (4) partial duplicates. Unreferenced subgroup analysis was defined as an article that did not disclose itself as a subgroup analysis or reference its main article. Unreferenced subgroup analyses are published following the main articles. We defined 4 subtypes: (1) subgroup of recruiting centers, in which only participants from a subset of recruiting centers are included; (2) subgroup of treatment groups, in which only participants from a subset of treatment groups are included; (3) subgroup of participants' characteristics, in which only participants with specific characteristics are included, for instance, the main article was on heart failure while an unreferenced subgroup analysis was on heart failure and diabetes; and (4) subgroup of recruiting centers and participants' characteristics.

Unreferenced republication was defined as an article that did not disclose itself as a replicate of the main article or reference the main article. A replicate means the duplicate reports identical participants and follow-up period as the main article. Unreferenced republications are published following the main articles.

Unreferenced interim analysis was defined as an article that did not disclose itself as an interim analysis or was not referenced by the subsequently published main article. We defined 3 subtypes: (1) interim report of recruitment, which reports an interim analysis on a subset of participants before recruitment is complete, usually the participants recruited in the early stage; (2) interim report of follow-up, which reports an interim analysis on all the participants but before the follow-up is complete, usually on short-term outcomes; and (3) interim report of both recruitment and follow-up, ie, an interim analysis on a subset of participants before recruitment and follow-up are complete.

A partial duplicate was an article that contained a portion of unique participants while sharing a portion of participants, eg, a subset of recruiting centers, with a main article that is not referenced. Partial duplicates were published after the main articles.

We also classified duplicates into cross-language duplicates and same-language duplicates. Cross-language duplicates referred to those published in a different language than the main articles, ie, the main article was published in Chinese and the duplicate in English, or vice versa. Same-language duplicates referred to those published in the same language as the main articles, ie, the main article and the duplicate were both published in Chinese or English. We hypothesized that the main articles with positive findings were more likely to have subsequent duplicates than those with negative findings.

### Statistical Analysis

#### Exposure

The exposure was the finding of a main article classified as positive or negative according to its primary outcome. If multiple primary outcomes were reported, the first one reported in the results section was selected. If no primary outcome was defined, we selected the primary outcome based on the following hierarchical order: (1) the first outcome used in the sample size calculation, (2) the first outcome defined in the study objectives, or (3) the first outcome reported in the results section.^28^ When the primary outcome was measured at multiple time points, we considered the last time point in the analysis.

We defined positive findings as those favoring the study hypothesis with statistical significance. For example, a superiority trial was positive when the experimental drug was significantly superior to the comparator. We defined negative findings as those not statistically significant or that did not support the study hypothesis.

#### Outcome and Effect Modifier

A main article had duplicates when at least 1 unreferenced subgroup analysis, 1 unreferenced republication, or 1 partial duplicate was identified from the same RCT. We hypothesized that the language of the main article was a possible effect modifier, ie, the association between the study findings and having subsequent duplicates might vary in the language of the main article.

#### Measurements of Associations

Duplicate publication bias was measured by relative risk (RR). An RR larger than 1.00 indicated that the main articles with positive findings were more likely to have subsequent duplicates than those with negative findings. The RRs were estimated using log binomial models with 4 covariates: language of the main article (Chinese vs English), sample size (<100 vs ≥100 participants), funding source (industry vs nonindustry), and number of recruiting centers (single vs multiple).^29,30,31^ An RCT was defined as funded by industry if at least 1 funder was from industry. An interaction term was included in the model to account for the language of the main article as an effect modifier. Statistical significance was defined as a 2-sided *P* < .05 for the main effect and *P* = .10 for the interaction. Data management and analysis were performed with SAS version 9.4 (SAS Institute Inc).

## Results

The search through trial registries and bibliographic databases was conducted from March 1 to August 31, 2019. A total of 470 of 891 (52.8%) eligible RCTs identified from trial registries had been published as journal articles. Fifty-five (11.7%) RCTs were found to have 75 duplicates, of which 48 (64.0%) were published in Chinese (Figure). None of the 27 English duplicates were labeled as duplicate publications by PubMed. Ten (18.2%) RCTs had multiple duplicates (range, 2 duplicates to 6 duplicates).

**Figure.**
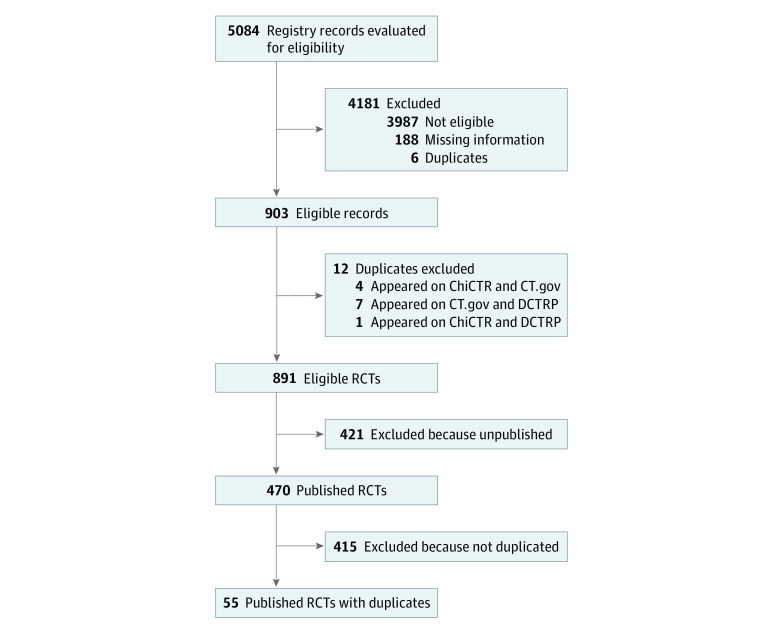
Study Flowchart Identifying Chinese-Sponsored Randomized Clinical Trials With Duplicates ChiCTR indicates Chinese Clinical Trial Registry; CT.gov, ClinicalTrials.gov; DCTRP, Drug Clinical Trial Registry Platform.

Fifty-three (70.7%) duplicates crossed languages, including 20 (26.7%) published in English (with the main article in Chinese) and 33 (44.0%) published in Chinese (with the main article in English). Twenty-two (29.3%) duplicates were published in the same language as their corresponding main articles; of these, 15 (20.0%) were published in Chinese and 7 (9.3%) in English (Table 1).

**Table 1.** Types of Duplicates^a^

Type	No. (%)						
	Cross language^b^			Same language^c^			Total
	Language of the main article		Total	Language of the main article		Total	
	Chinese	English		Chinese	English		
**Unreferenced interim analysis**							
Interim report of recruitment	0	9 (12.0)	9 (12.0)	2 (2.7)	1 (1.3)	3 (4.0)	12 (16.0)
Interim report of follow-up	0	2 (2.7)	2 (2.7)	0	0	0	2 (2.7)
Interim report of both recruitment and follow-up	0	1 (1.3)	1 (1.3)	0	0	0	1 (1.3)
Total	0	12 (16.0)	12 (16.0)	2 (2.7)	1 (1.3)	3 (4.0)	15 (20.0)
**Unreferenced republication**							
No mention of main article	15 (20.0)	10 (13.3)	25 (33.3)	6 (8.0)	2 (2.7)	8 (10.7)	33 (44.0)
**Unreferenced subgroup analysis**							
Subgroup of recruitment centers	2 (2.7)	1 (1.3)	3 (4.0)	4 (5.3)	0	4 (5.3)	7 (9.3)
Subgroup of treatment groups	0	3 (4.0)	3 (4.0)	1 (1.3)	0	1 (1.3)	4 (5.3)
Subgroup of participants' characteristics	0	2 (2.7)	2 (2.7)	0	2 (2.7)	2 (2.7)	4 (5.3)
Subgroup of centers’ and participants' characteristics	0	2 (2.7)	2 (2.7)	0	0	0	2 (2.7)
Unclear	2 (2.7)	3 (4.0)	5 (6.7)	2 (2.7)	1 (1.3)	3 (4.0)	8 (10.7)
Total	4 (5.3)	11 (14.7)	15 (20.0)	7 (9.3)	3 (4.0)	10 (13.3)	25 (33.3)
**Partial duplicate**							
Shared subsets	1 (1.3)	0	1 (1.3)	0	1 (1.3)	1 (1.3)	2 (2.7)
Total	20 (26.7)	33 (44.0)	53 (70.7)	15 (20.0)	7 (9.3)	22 (29.3)	75 (100.0)

^a^ Duplicates were classified as cross-language duplicates or same-language duplicates. A main article had duplicates when at least 1 unreferenced subgroup analysis, 1 unreferenced republication, or 1 partial duplicate was identified from the same randomized clinical trial.

^b^ Cross-language duplicates referred to those articles published in a different language than the main articles, ie, the main article was published in Chinese and the duplicate in English, or vice versa.

^c^ Same-language duplicates referred to those articles published in the same language as the main articles, ie, the main article and the duplicate were both published in Chinese or English.

The characteristics of RCTs having duplicates were generally similar to RCTs without duplicates. Among 55 RCTs having duplicates, 10 (18.8%) were sponsored by industry (as opposed to nonindustry), 25 (47.2%) recruited fewer than 100 participants, 13 (24.5%) were conducted at 1 recruiting center only, and 38 (71.7%) reported positive findings. Among 415 RCTs without duplicates, 83 (19.9%) were sponsored by industry, 162 (38.9%) recruited fewer than 100 participants, and 135 (32.4%) were conducted at 1 recruiting center. Two RCTs were excluded from the analysis because their exposure status could not be determined (no between-group comparison was reported). In the remaining 413 RCTs without duplicates, 292 (70.4%) reported positive findings.

### Duplicate Types

The most prevalent duplicate type was unreferenced republication of the main article (Table 1). Thirty-three (44.0%) of 75 duplicates fit this type, including all 4044 participants reported in the main articles. Of these, 25 (75.8%) duplicates crossed languages, including 15 (45.5%) published in English (with the main article in Chinese) and 10 (30.3%) in Chinese (with the main article in English).

Twenty-five (33.3%) duplicates were unreferenced subgroup analyses, of which 7 (28.0%) were subgroups of centers, 4 (16.0%) were subgroups of treatment groups, 4 (16.0%) were subgroups of participants' characteristics, 2 (8.0%) were subgroups of both centers and participants' characteristics, and 8 (32%) were unclear owing to lack of sufficient information. The 25 unreferenced subgroup analyses included 2193 (66.6%) of 3295 participants reported in the main articles. Fifteen (60.0%) duplicates crossed languages, including 11 (44.0%) published in Chinese (with the main article in English) and 4 (16.0%) in English (with the main article in Chinese).

Fifteen (20.0%) of 75 duplicates were unreferenced interim analyses, of which 12 (80.0%) reported early-stage recruitment, 2 (13.3%) reported early-stage follow-up, and 1 (6.7%) reported a mixture of early-stage recruitment and follow-up. The 15 unreferenced interim analyses included 1125 of 2468 participants (45.6%) reported in the main articles. Twelve duplicates (80.0%) crossed languages, all of which were in Chinese (with the main articles in English).

There were 2 partial duplicates (2.7%), of which 1 crossed languages (the main article in Chinese) and the other was the same language as the main article (in Chinese).

### Duplicate Publication Bias

We did not find evidence supporting the language of the main article as an effect modifier (χ^2^ = 1.60, *P* = .21). After adjusting for covariates, when published in Chinese, the main articles with positive findings were 2.48 (95% CI, 1.08-5.71) times more likely to have subsequent duplicates than RCTs with negative findings. There was no evidence supporting a similar bias when the main articles were published in English (RR, 0.99; 95% CI, 0.31-3.13). The main articles published in Chinese were 8.03 (95% CI, 3.91-16.46) times more likely to have subsequent duplicates than those published in English. None of other covariates reached statistical significance (Table 2).

**Table 2.** Factors Associated With Duplicate Publication Bias

Factor	Comparison	Relative risk (95% CI)	*P* value
Nature of findings of the main article			
The main article was in Chinese	Positive vs negative	2.48 (1.08-5.71)	.03
The main article was in English	Positive vs negative	0.99 (0.31-3.13)	>.99
Language of the main article	Chinese vs English	8.03 (3.91-16.46)	<.001
Sample size	≥100 vs <100	1.07 (0.54-2.13)	.85
Funding	Nonindustry vs industry	1.39 (0.59-3.26)	.45
No. of recruiting centers	Single center vs multiple centers	1.58 (0.71-3.50)	.26

## Discussion

Findings of this cohort study suggest that duplicates continue to populate the medical literature, especially studies published in more than 1 language that are challenging to detect. Whether the occurrence of duplicates owes to a lack of awareness on behalf of trial investigators (some investigators may produce duplicates unaware of the inappropriateness) or to deliberate production is unclear.

Aside from research integrity and legal considerations, the presence of duplicates carries the potential to distort evidence when an article may be counted more than once in systematic reviews.^3,4^ Currently there is a gap between English and Chinese literature: most Chinese RCTs are published in Chinese and only indexed in Chinese bibliographic databases, and very few Chinese journals are covered by major English bibliographic databases.^14,16^ At the same time, English-language systematic reviews, which should bridge the language gap, rarely search Chinese bibliographic databases and subsequently do not include RCTs published in Chinese.^32^ Under these circumstances, the cross-language duplicates raise serious concerns, especially when the main articles are published in Chinese and the duplicates are in English, because English duplicates may selectively report subsets of participants from the main articles with positive findings or larger effect sizes. Although this type was only present in 5% of all duplicates in our sample, reviewers should be vigilant considering the sheer quantity of RCTs being conducted in China.

In contrast, simply excluding the Chinese literature is inappropriate as it may lead to other negative consequences such as missing relevant RCTs.^14^ Moreover, language bias has been found among RCTs from mainland China; excluding the Chinese literature may increase the risk of overestimating treatment effects, as RCTs with positive findings are more likely to be published in English than those with negative findings.^20^

The coverage of Chinese journals by major English bibliographic databases has been increasing gradually while increasing numbers of systematic reviews have recently begun to include Chinese bibliographic databases to reduce bias.^18^ In theory, expansion of the overlap between English and Chinese literature may help increase the possibility of unintentional inclusion of duplicates in systematic reviews. While an unreferenced republication may be detected by a meticulous reviewer or editor based on identical study participants and results as the main article, unreferenced subgroup analyses and unreferenced interim analyses can be much more challenging to detect owing to different participants or results from the main articles.

We found that the RCTs in which the main articles were published in Chinese were more likely to have subsequent duplicates than those in which the main articles were published in English, a phenomenon that might be explained by the desire to publish research in English journals with higher impact factors and reputation. Although the statistical test on the effect modification by the language of the main article was not statistically significant, duplicate publication bias only existed when the main articles were published in Chinese. This finding suggests that the language of publication may have the potential to modify the duplicate publication bias; our limited sample size is likely a factor associated with the negative result of the statistical test.

In our study, we could not determine the duplication status of some articles owing to a lack of key information or inconsistencies between trial registries and published journal articles.^33,34,35^ Improving the data quality of trial registries and the reporting quality of journal articles could enable reviewers to identify duplicates more efficiently.

### Limitations

This study has limitations. First, our sample size was limited and a larger sample of RCTs would enable us to better quantify the association between language and bias. Second, we applied strict rules with high specificity to identify duplicates at the cost of sensitivity. Consequently, we may have missed some duplicates, especially those that are substantially inconsistent with the main articles or lacking information for classification. Therefore, the actual prevalence of duplicates may be higher than our estimate and there may be duplicate types that we did not identify. Third, because less than 15% of RCTs from mainland China were registered,^36^ RCTs in trial registries may not be representative of all RCTs from China. Our conclusions may not be generalizable to other settings. Fourth, although we found evidence supporting the existence of duplicate publication bias, further study is warranted to determine the extent to which duplicates, especially cross-language duplicates, may affect the findings of individual systematic reviews. In addition, we selected only RCTs from mainland China as our study sample. The prevalence, types, and biases regarding duplicates may vary in other languages. Nonetheless, we believe our study may help inform future research on RCTs in other languages.

## Conclusions

Most duplicate publications of RCTs conducted in mainland China in this study were cross-language duplicates and unreferenced republications of the main articles. Duplicate publication bias exists when the main articles were published in Chinese, potentially misleading readers and compromising journals and evidence synthesis.

## Notes

Supplement. **eMethods.** List of Trial Registries and Bibliographic Databases **eTable 1.** Strategies to Search Bibliographic Databases **eTable 2.** Terms to Search Bibliographic Databases **eTable 3.** Criteria to Match Journal Articles with Registry Records **eAppendix.** An Example of Search Terms
