# Supplementary material for: Assessment of Duplicate Publication of Chinese-Sponsored Randomized Clinical Trials
Source: JAMA Netw Open. 2020 Dec 3;3(12):e2027104. doi: 10.1001/jamanetworkopen.2020.27104 (PMC7716193; doi:10.1001/jamanetworkopen.2020.27104)
Supplement: Supplement. — eMethods. List of Trial Registries and Bibliographic Databases eTable 1. Strategies to Search Bibliographic Databases eTable 2. Terms to Search Bibliographic Databases eTable 3. Criteria to Match Journal Articles with Registry Records eAppendix. An Example of Search Terms [file jamanetwopen-e2027104-s001.pdf]

Supplemental Online Content

Jia Y, Huang D, Wen J, et al. Assessment of duplicate publication of Chinese-sponsored randomized clinical trials. *JAMA Netw Open*. 2020;3(12):e2027104. doi:10.1001/jamanetworkopen.2020.27104

eMethods. List of Trial Registries and Bibliographic Databases

eTable 1. Strategies to Search Bibliographic Databases

eTable 2. Terms to Search Bibliographic Databases

eTable 3. Criteria to Match Journal Articles with Registry Records

eAppendix. An Example of Search Terms

This supplemental material has been provided by the authors to give readers additional information about their work.

© 2020 Jia Y et al. *JAMA Network Open*.

eMethods. List of Trial Registries and Bibliographic Databases

Trial Registries

Primary Registries Recognized by the World Health Organization

- Australian New Zealand Clinical Trials Registry (ANZCTR)
- Brazilian Clinical Trials Registry (ReBec)
- Chinese Clinical Trial Registry (ChiCTR)
- Clinical Research Information Service (CRiS), Republic of Korea
- Clinical Trials Registry - India (CTRI)
- Cuban Public Registry of Clinical Trials (RPCEC)
- EU Clinical Trials Register (EU-CTR)
- German Clinical Trials Register (DRKS)
- Iranian Registry of Clinical Trials (IRCT)
- ISRCTN
- Japan Primary Registries Network (JPRN)
- Lebanese Clinical Trials Registry (LBCTR)
- Thai Clinical Trials Registry (TCTR)
- The Netherlands National Trial Register (NTR)
- Pan African Clinical Trial Registry (PACTR)
- Peruvian Clinical Trial Registry (REPEC)
- Sri Lanka Clinical Trials Registry (SLCTR)
- ClinicalTrials.gov

Trial Registry by the China Food and Drug Administration

- Drug Clinical Trial Registry Platform

Bibliographic Databases

- PubMed
- Embase
- the Cochrane Central Register of Controlled Trials (CENTRAL)
- the China National Knowledge Infrastructure (CNKI)
- SinoMed
- the VIP information
- the Wanfang Data

© 2020 Jia Y et al. *JAMA Network Open*.

eTable 1. Strategies to Search Bibliographic Databases

Strategy 1	Single-Center and Multi-Center RCTs	Registration Number
Strategy 2	Single-Center and Multi-Center RCTs	PI's Name AND PI's Affiliation AND (Disease OR Drug) AND Study Start Date
Strategy 3	Multi-Center RCTs	Recruitment Facilities AND Disease AND Drug AND Study Start Date

Abbreviations

PI: Principal Investigator; RCT: Randomized Clinical Trials.

We used all three strategies for each record from trial registries to capture as many publications as possible. When constructing the search terms, we used the logical operator 'OR' to combine all possible spelling variations and synonyms for the disease and the drug, as listed in eTable 3, together to maximize sensitivity.

© 2020 Jia Y et al. *JAMA Network Open*.

eTable 2. Terms to Search Bibliographic Databases

Database	Identifier	Source of Subjects	Source of Keywords
English	Disease	MeSH<br>Emtree	Registry records<br>PubMed entry terms<br>Embase synonyms
	Drug	MeSH<br>Emtree	Registry records<br>PubMed entry terms<br>Embase synonyms
Chinese	Disease	MeSH (Chinese version)	Registry records<br>ICD-10 (Chinese version)<br>ICD-9 (Chinese version)<br>Three doctors from PUMC
	Drug	MeSH (Chinese version)	Registry records<br>China FDA website for drug trade names and compound names<br>Three pharmacists from PUMC

Abbreviations

MeSH: Medical Subject Headings (the control vocabulary from Medline); Emtree: Embase Subject Headings (the control vocabulary from Embase); ICD-9: The International Classification of Diseases, 9th Revision; ICD-10: The International Classification of Diseases, 10th Revision; PUMC: Peking Union Medical College; China FDA: China Food and Drug Administration

© 2020 Jia Y et al. *JAMA Network Open*.

eTable 3. Criteria to Match Journal Articles with Registry Records

**Criteria**
Similar Eligibility Criteria & Interventions, Same Registration Number
Similar Eligibility Criteria & Interventions, Same Ethics Committee Approval Number
Similar Eligibility Criteria & Interventions, Same Funding Identification
Consistent Eligible Criteria & Interventions, Similar Sample Size & Overlapped Study Period
Consistent Eligible Criteria & Interventions, Identical Sample Size
Consistent Eligible Criteria & Interventions, Identical Study Period

© 2020 Jia Y et al. *JAMA Network Open*.

eAppendix. An Example of Search Terms

Below is an example of search terms for the RCT with registration number ChiCTR-IPR-16008578. We listed the search terms for PubMed.

First, we searched the registration number:

"ChiCTR-IPR-16008578"

Second, we applied Strategy 2:

#1 Author: "Liu Y"[AU] OR "Ying L"[AU]

#2 Affiliation: "Tianjin"[AD] OR "Tian Jin"[AD]

#3 Disease: "Diabetes Mellitus"[MeSH] OR "Diabetes Mellitus"[tw] OR "diabetes mellitus"[tw] OR "diabetes"[tw] OR "diabetic"[tw]

#4 Drug: "Liraglutide"[MeSH] OR "Liraglutide"[tw] OR "liraglutide"[tw] OR "204656-20-2"[tw] OR "nn 2211"[tw] OR "nn2211"[tw] OR "NN-2211"[tw] OR "nnc 90 1170"[tw] OR "nnc 90-1170"[tw] OR "nnc90 1170"[tw] OR "nnc90-1170"[tw] OR "saxenda"[tw] OR "victoza"[tw] OR "Metformin"[MeSH] OR "Metformin"[tw] OR "metformin"[tw] OR "1, 1 dimethylbiguanide"[tw] OR "1115-70-4"[tw] OR "657-24-9"[tw] OR "apophage"[tw] OR "aron"[tw] OR "benofomin"[tw] OR "dabex"[tw] OR "denkaform"[tw] OR "deson"[tw] OR "dextin"[tw] OR "diabetase"[tw] OR "diabetformin"[tw] OR "diabetmin"[tw] OR "diabetosan"[tw] OR "diabex"[tw] OR "diafat"[tw] OR "diaformin"[tw] OR "diaformina"[tw] OR "diametin"[tw] OR "diamin"[tw] OR "dianben"[tw] OR "diformin"[tw] OR "dimefor"[tw] OR "dimethylbiguanide"[tw] OR "Dimethylbiguanidine"[tw] OR "dimethyldiguanide"[tw] OR "Dimethylguanylguanidine"[tw] OR "dmgg"[tw] OR "dybis"[tw] OR "eraphage"[tw] OR "espa-formin"[tw] OR "euform retard"[tw] OR "fluamine"[tw] OR "flumamine"[tw] OR "fornidd"[tw] OR "fortamet"[tw] OR "glafornil"[tw] OR "glibudon"[tw] OR "glifage"[tw] OR "gliguanid"[tw] OR "glucaminol"[tw] OR "glucofage"[tw] OR "glucofago"[tw] OR "glucoform"[tw] OR "glucoformin"[tw] OR "glucohexal"[tw] OR "glucoless"[tw] OR "glucomet"[tw] OR "glucomin"[tw] OR "glucomine"[tw] OR "gluconil"[tw] OR "glucophage"[tw] OR "glucophage-mite"[tw] OR "glucostop"[tw] OR "glucotika"[tw] OR "gludepatic"[tw] OR "glufor"[tw] OR "gluformin"[tw] OR "glukophage"[tw] OR "glumeformin"[tw] OR "glumet"[tw] OR "glumetza"[tw] OR "glupa"[tw] OR "glustress"[tw] OR "glyciphage"[tw] OR "glycomet"[tw] OR "glycon"[tw] OR "glycoran"[tw] OR "glyformin"[tw] OR "glymet"[tw] OR "haurymellin"[tw] OR "hipoglucin"[tw] OR "i-max"[tw] OR "islotin"[tw] OR "juformin"[tw] OR "la 6023"[tw] OR "la6023"[tw] OR "maformin"[tw] OR "meglucon"[tw] OR "meguan"[tw] OR "melbin"[tw] OR "melformin"[tw] OR "mellittin"[tw] OR "merckformin"[tw] OR "mescorit"[tw] OR "metaformin"[tw] OR "metfogamma"[tw] OR "metforal"[tw] OR "metformax"[tw] OR "Metformin HCl"[tw] OR "metformine"[tw] OR "methformin"[tw] OR "metiguanide"[tw] OR "metomin"[tw] OR "metphormin"[tw] OR "miformin"[tw] OR "neoform"[tw] OR "nndg"[tw] OR "reglus-500"[tw] OR "riomet"[tw] OR "risidon"[tw] OR "siamformet"[tw] OR "siofor"[tw] OR "thiabet"[tw] OR "vimetrol"[tw] OR "walaphage"[tw]

#5 Study Date: "2012-04-01"[Date - Publication]:"3000"[Date - Publication]

#6 Registration Number: "ChiCTR-IPR-16008578"

The full search terms are:

#1 AND #2 AND (#3 OR #4) AND #5 NOT #6

© 2020 Jia Y et al. *JAMA Network Open*.
